# Fatigue Crack Growth Rates and Crack Tip Opening Loads in CT Specimens Made of SDSS and Manufactured Using WAAM

**DOI:** 10.3390/ma17081842

**Published:** 2024-04-17

**Authors:** Andrew Sales, Aditya Khanna, James Hughes, Ling Yin, Andrei Kotousov

**Affiliations:** 1 School of Electrical and Mechanical Engineering, The University of Adelaide, Adelaide, SA 5005, Australia; james.m.hughes@adelaide.edu.au (J.H.); ling.yin@adelaide.edu.au (L.Y.); 2 AML3D Limited, 35 Woomera Avenue, Edinburgh, SA 5111, Australia; 3 School of Mechanical & Mining Engineering, The University of Queensland, Brisbane, QLD 4072, Australia; aditya.khanna@uq.edu.au

**Keywords:** fatigue, crack tip opening load, compact tension (CT) specimen, WAAM, super duplex stainless steel, compliance-based method

## Abstract

Additive manufacturing offers greater flexibility in the design and fabrication of structural components with complex shapes. However, the use of additively manufactured parts for load-bearing structural applications, specifically involving cyclic loading, requires a thorough investigation of material fatigue properties. These properties can be affected by many factors, including residual stresses and crack tip shielding mechanisms, which can be very different from those of conventionally manufactured materials. This research focuses on super duplex stainless steels (SDSSs) fabricated with wire arc additive manufacturing (WAAM) and investigates their fatigue crack growth rates and the net effect of crack tip shielding mechanisms. Using the compliance-based method, we measured crack tip opening loads in compact tension (CT) specimens with cracks propagating longitudinally and transversely to the WAAM deposition direction. It was found that fatigue crack growth rates were very similar in both directions when correlated by the effective stress intensity factor range. However, the differences in crack tip opening loads explain a quite significant influence of the deposition direction on the fatigue life.

## 1. Introduction

Super duplex stainless steels (SDSSs) are commonly used in marine and offshore applications for pressure containment and load bearing structures subject to corrosive and fatigue-prone environments [1,2,3]. SDSSs have impressive mechanical properties and microstructural characteristics, resulting in a unique combination of high strength, fracture toughness, corrosion resistance and resistance to stress corrosion cracking for cast, forged, welded and billet-processed materials [1,4,5,6,7,8,9,10]. In recent years, gas metal arc welded additive manufacturing (GMA-DED) or wire arc additive manufacturing (WAAM) has seen a commercial uptake in industry due to reduced lead times in processing and the ability to manufacture near net shapes [7,11,12,13,14,15]. 

Although research has been conducted on various grades of duplex stainless steels and has increased in recent years, the majority of these studies, which are supportive of the commercial use of SDSSs fabricated with WAAM for load-bearing structures, are predominantly limited to investigations of basic material properties and microstructure characterisation [7,16,17,18]. The fatigue behaviour of SDSSs in terms of S-N diagrams have become recently available [7,16,19,20]. In particular, these studies reported a significant anisotropy of the fatigue life, which strongly depends on the orientation of fatigue test specimens with respect to the WAAM deposition direction. The reason for the anisotropy is not clear, and the observed differences in the fatigue life are often attributed to the material anisotropy, residual stresses, as well as manufacturing defects, which normally have a preferable orientation in components fabricated using WAAM [21].

The utilisation of the contemporary design concepts and maintenance strategies, such as the damage tolerance concept, also requires the knowledge of fatigue crack growth (FCG) rates. Past studies reported the FCG rates for conventionally manufactured duplex steels [22,23]. Some key studies of FCG have been conducted for medium to high strength steel grades manufactured using WAAM [24,25,26]. However, little is known about the fatigue properties, specifically FCG rates, of SDDSs manufactured with WAAM. Stützer et al. (2019) reviewed stainless steels including SDSSs fabricated using WAAM [18] and concluded that the fatigue properties of these steels are inherently different when compared with the same materials processed conventionally (casting, forging, and billeting). Therefore, further research is needed to understand the fatigue properties and behaviours under cyclic loading for SDSSs manufactured using WAAM [19,20,27].

This paper addresses the lack of FCG data through investigating crack propagation rates and crack tip opening loads in CT specimens when a fatigue crack propagates longitudinally and transversely to the WAAM deposition direction. FCG rates and crack tip opening load ratios are reported for a wide range of loading conditions. 

## 2. Materials and Methods

A large test wall was manufactured through a WAAM process using wire grade ER2594 SDSS for the feedstock. Layers were deposited with dwell times and parameters as per previous research by the authors [20]. The deposition parameters are listed in Table 1. Once the large test wall was manufactured, tensile specimens were cut and machined in compliance with the tensile testing standard ASTM E8/E8M-16a [28] to determine the material properties. Furthermore, cross-sectional specimens were cut and etched using both Beraha II and 10% KOH for metallographic examination to determine the ferrite and austenite balance using ASTM E1245 [29].

**Figure 1:**
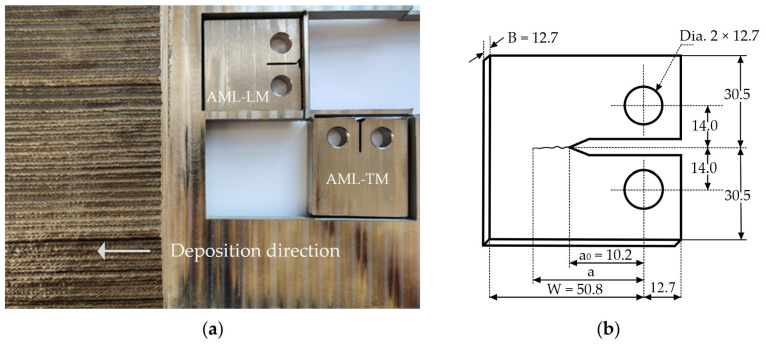
Specimen fabrication. (**a**) WAAM manufactured test wall showing C(T) specimens with notches longitudinal and transverse to the deposition direction; (**b**) dimensions of C(T) specimens.

The test specimens for fatigue testing were cut from the WAAM printed test wall using water jet cutting so as to not affect the specimens thermally. Then, the cut specimens were milled perpendicularly to the WAAM deposition direction, according to ASTM E647 dimensional requirements as shown in Figure 1. The machined notches shown in Figure 1a were such that one had a crack direction oriented longitudinal to the deposition direction (AML-LM) and one was in transverse to the deposition direction (AML-TM). Each specimen had a thickness of 12.7 mm, a nominal width of 63.5 mm, and a height of 61 mm, as shown in Figure 1b.

Fatigue testing was performed on one specimen only per direction. This was considered sufficient for comparative purposes. However, in other situations, where the data may be used for design calculations or life prediction purposes, multiple replicate tests are desirable to quantify variability in the fatigue crack growth rates between specimens cut from different regions of the same test wall or specimens cut from different test walls fabricated using the same process parameters. Such replicate testing is outside the scope of the present study, which is comparative in nature.

**Figure 2:**
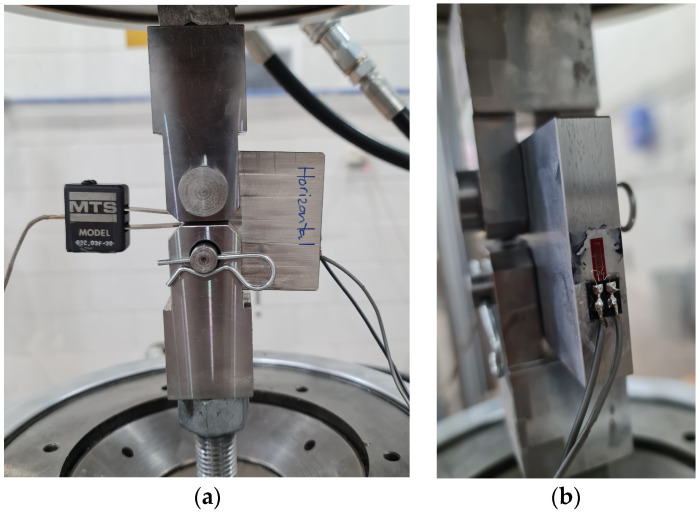
Instrumentation and testing C(T) specimens. (**a**) Shows the specimen with clip gauge (**b**) shows the close look of the back strain gauge.

The specimens were instrumented with resistive back-face strain gauges which were placed directly behind the starter notch and a clip gauge placed in the crack mouth as shown in Figure 2. Both specimens were pre-cracked under tension–tension loading with a maximum load of $P_{max}=$ 8.5 kN and a minimum load of $P_{min}=$ 0.5 kN (stress ratio R~0.06). For the AML-LM specimen (crack path longitudinal to the deposition direction), 550,000 cycles were required to achieve a crack extension of approximately 2.1 mm. For the AML-TM specimen (crack path transverse to the deposition direction), 288,000 cycles were applied and a crack extension of approximately 2.2 mm was achieved. The stress intensity factor range ∆K during pre-cracking was around 14 MPa m^1/2^.

**Figure 3:**
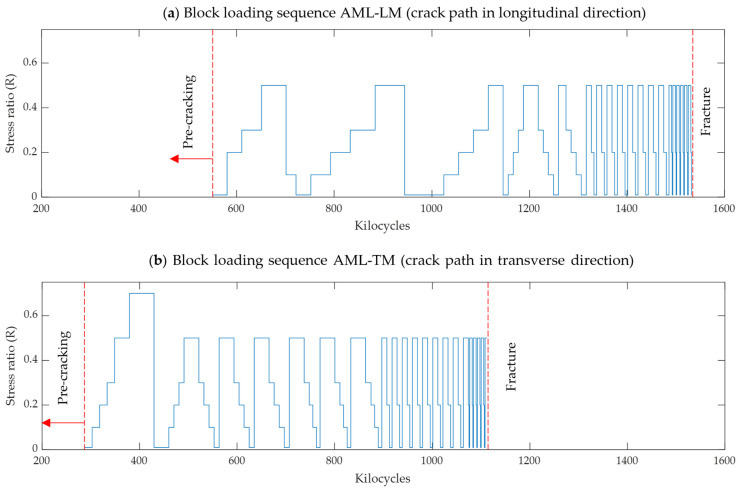
Block loading sequence for tests performed at constant maximum load and variable minimum load (stress ratio). (**a**) AML-LM specimen; (**b**) AML-TM specimen.

After pre-cracking, block loading was repeatedly applied to the specimens until failure. The maximum load was kept constant for the entire duration of the test at $P_{max}=$ 9.5 kN. The minimum load was stepped between 0.095 kN (R= 0.01) and 4.75 kN (R= 0.5). The number of cycles applied per load block (constant R) was adjusted throughout the test to achieve a roughly uniform spacing of the data points on the logarithmic da/dN versus ΔK curves. For normalised crack lengths in the range of 0.2 < a/W < 0.4, the block loading nominally consisted of 5 blocks corresponding to R= 0.01, 0.1, 0.2, 0.3, and 0.5 (specimen AML-TM also subjected to one block at R = 0.7). At longer normalised crack lengths (0.4 < a/W < 0.8), the loading was stepped only 3 times, corresponding to blocks of R= 0.01, 0.2, and 0.5. The full load history applied to both specimens (after pre-cracking until fracture) is summarised in Figure 3. The cycles to failure (fatigue lives) were approximately 1.53 million cycles for AML-LM and 1.11 million cycles for AML-TM. Caution must be applied when comparing the fatigue lives, since the load histories applied to the two specimens were similar but not identical.

Compliance-based methods were used to determine the crack length, as well as the crack closure (opening) loads for the entire duration of the test. For compliance calculation, the back-face strain gauge reading was preferred over the crack mouth opening displacement measurements. The latter is known to be error-prone for pin loaded specimens, such as the CT specimens used in the present study. The details of the compliance-based methods are not presented here since these are standardised in ASTM E647 [30] and reviewed extensively in past studies [31,32,33,34,35,36].

The back-face compliance relationship used to determine the crack length is [30]

(1) $$a/W=1.0033-2.35\lambda +1.3694\lambda^{2}-15.294\lambda^{3}+63.182\lambda^{4}-74.42\lambda^{5}$$

where $\lambda =1/\left(1+\sqrt{A}\right)$ and $A=E^{*}\cdot B\cdot W\cdot C$. The specimen thickness B and width W are defined in Figure 1b. The plane strain Young’s modulus is defined as $E^{*}=E/\left(1-\nu^{2}\right)$, where E = 190 GPa and ν = 0.28 were determined from mechanical tests. The open crack compliance $C=\left|\varepsilon /P\right|$ was calculated using the portion of the unloading curve which spanned from 0.7$P_{max}$ to 0.95$P_{max}$ to reduce any transient effects at low strain rates and to ensure that the load applied was high enough such that the crack was fully open. A linear regression analysis was used to determine the compliance by plotting the strain ε against the corresponding load, P.

The crack opening loads were obtained using the compliance offset method. This differential method is highly sensitive to external noise in the strain and load signals. Hence, some form of noise filtering is almost always required to obtain acceptable signal to noise ratios. Various noise filtering techniques have been proposed in the past, such as moving average filter, lowpass filter, etc. Guided by previous in-depth studies on crack closure measurement [33,34,35,36], we applied a third order lowpass Butterworth filter to the raw load and strain signals. A sufficiently high cut-off frequency equal to 10 times the loading frequency was selected to filter out high-frequency electrical noise without altering the nonlinear response generated by crack closure. While the fatigue test was nominally performed at a loading frequency of 10 Hz, the test frequency was intermittently reduced to 2 Hz to capture high resolution data for crack opening load estimation. Reducing the loading frequency was necessary to increase the data fidelity in the opening load estimate. As demonstrated by the authors in previous studies, this practical constraint is imposed by the type of sensor used (resistive strain gauge in this case) and can be resolved by using advanced sensors with a lower noise floor [34].

**Figure 4:**
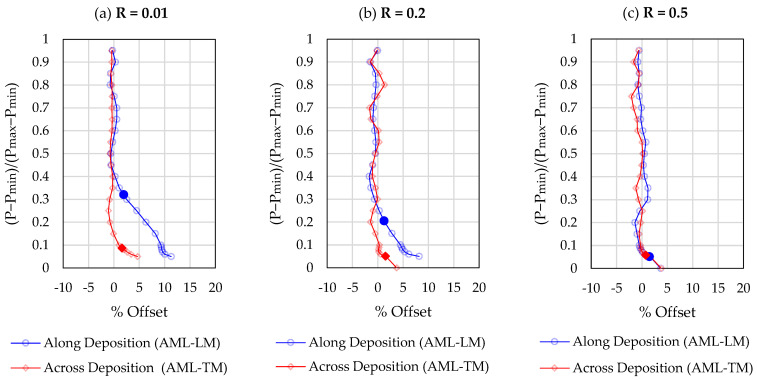
Representative compliance offset curves ata/W = 0.25. The solid symbols represent the crack opening load estimate. (**a**) R = 0.01; (**b**) R = 0.2; (**c**) R = 0.5.

**Figure 5:**
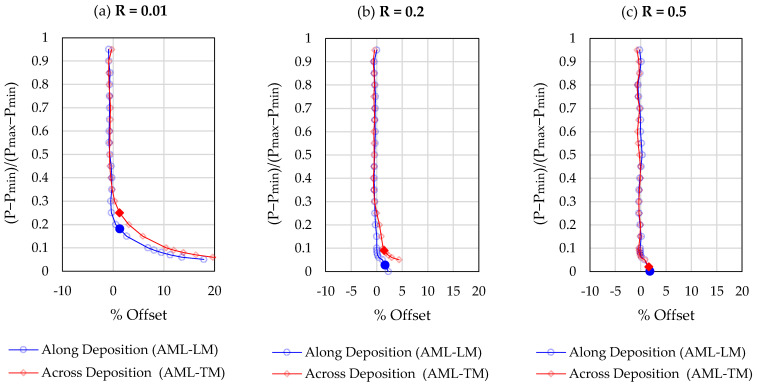
Representative compliance offset curves at a/W = 0.60. The solid symbols represent the crack opening load estimate. (**a**) R = 0.01; (**b**) R = 0.2; (**c**) R = 0.5.

As per ASTM recommendations, a 10% span and 5% shift was used to segment the loading portion of the stress cycle and estimate the compliance offset relative to the open crack compliance. The opening load corresponds to a 2% compliance offset. In accordance with previous studies, two modifications were made to the standardised compliance offset method described in ASTM E647. The first modification pertains to high load ratio sequences for which the opening load is expected to be close to or equal to the minimum load. Using the recommended span and shift values, the standardised compliance offset method cannot determine the opening loads $P_{op}<P_{min}+0.05\Delta P$. Following the extrapolation method recommended by Chung and Song (2009) [33], the compliance offset curve is extrapolated to $P_{min}$ in situations where the opening load cannot be identified using the standard method. The second modification pertains to the hysteresis in the load loop. Due to hysteresis, the experimentally measured compliance offset at or near the maximum load is non-zero. Hence, the 2% compliance offset is taken relative to the mean compliance offset in the upper portion of the loading cycle [35]. Representative compliance offset curves for a “single” load cycle are shown at two different normalised crack lengths (a/W = 0.25, a/W = 0.6) in Figure 4 and Figure 5, respectively. Compliance changes are magnified for longer cracks, hence a reduced scatter is observed in the results obtained in Figure 5, as compared to Figure 4. A strong dependence of the crack opening load on the stress ratio (R) and crack length (a/W) is noted. These dependencies are investigated in greater detail in later sections. 

## 3. Results

This section describes the main outcomes of the experimental study in terms of fatigue life of the specimens, crack growth rates versus the stress intensity factor range and the crack tip opening ratio as a function of the R-ratio. Further, we correlate the crack growth rates for different R-ratios versus the effective stress intensity factor, $K_{eff}$, which accounts for the crack closure effect on the FCG rates.

### 3.1. Mechanical and Microstructural Properties 

Prior to proceeding with the fatigue testing, several tests were performed to establish the basic mechanical and microstructure properties. Microstructure examination resulted in a ferrite–austenite phase balance of approximately 36% ferrite and 64% austenite. The tensile yield stresses were approximately 678 MPa and 652 MPa for both longitudinal and transverse directions, respectively, with both results being consistent with the authors previous experiments [19,20] as well as similar research from other authors [4,7,8] for the same material and fabrication parameters. 

### 3.2. Fatigue Crack Growth Curves 

**Figure 6:**
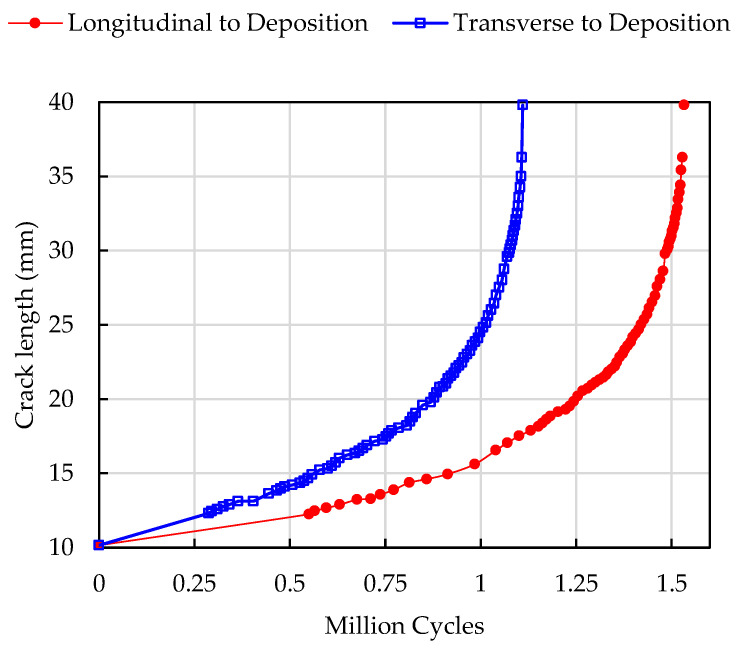
Fatigue crack growth curves for fatigue crack propagating longitudinally and transversely to the deposition direction.

Figure 6 shows the crack growth curves obtained for the applied load sequences as in Figure 3. In agreement with previous experimental studies on additively fabricated components, the fatigue life was strongly dependent on the orientation. The specimen with the notch (and fatigue crack propagation) longitudinal to (along) the deposition direction (AML-LM) had approximately a 50% longer fatigue life compared to the case when fatigue crack propagates transverse to (across) the deposition direction (AML-TM). The analogous difference was observed for smooth fatigue specimens in previous studies focusing on obtaining S-N diagrams [19,20]. Contrary to this, the specimen orientation was found to have almost no influence on fracture toughness, with both specimens failing when the fatigue crack reached a length of approximately 40 mm. This final length corresponds to a fracture toughness of 130 MPa m^1/2^, which correlates with the estimates of $K_{Ic}$ obtained for SDSS fabricated with conventional manufacturing methods (cast, rolled plate and welded) [10,36,37,38,39]. 

One important implication of the observed fatigue life anisotropy is in the enhancement of fatigue life at the design and fabrication stages. To prolong the fatigue life of components made of materials with anisotropic fatigue properties, one can align the material direction with the longest fatigue life perpendicular to the maximum applied cyclic stresses. In the case of WAAM, the longitudinal deposition direction has the longest fatigue life; therefore, it will be beneficial to fabricate structural components loaded by cyclic stress in such way that the deposition direction is perpendicular to the expected maximum cyclic stress during operation [20]. 

This section describes the standard fatigue properties in terms of the crack growth rate per cycle (da/dN) versus the stress intensity factor range (∆K), for different R-ratios and two crack propagation directions. These standard properties are often required for the design, evaluation and maintenance scheduling purposes as described in the Introduction. The stress intensity factor range is defined as

(2) $$∆K=K_{max}-K_{min},$$

where $K_{max}$ and $K_{min}$ are the maximum and minimum values of the stress intensity factor (SIF) in a loading cycle, respectively. The latter corresponds to the maximum, $P_{max}$, and minimum, $P_{min}$, loads in the cycle, respectively. For standard compact tension specimens, the SIF can be obtained in terms of the applied load as follows [33].

(3) $$K=\frac{P}{B\sqrt{W}}\frac{\left(2+\alpha \right)}{{\left(1-\alpha \right)}^{3/2}}\left(0.886+4.64\alpha -13.32\alpha^{2}+14.72\alpha^{3}-5.6\alpha^{4}\right).$$

The above expression is valid for α=a/W>0.2 and the geometric parameters are defined in Figure 1b.

### 3.3. Fatigue Crack Growth Rates

The stress ratio (R-ratio) can be expressed in terms of the maximum, $P_{max}$, and minimum, $P_{min}$, loads in the cycle, or the corresponding SIFs as follows:

(4) $$R=\frac{P_{min}}{P_{max}}=\frac{K_{min}}{K_{max}}.$$

**Figure 7:**
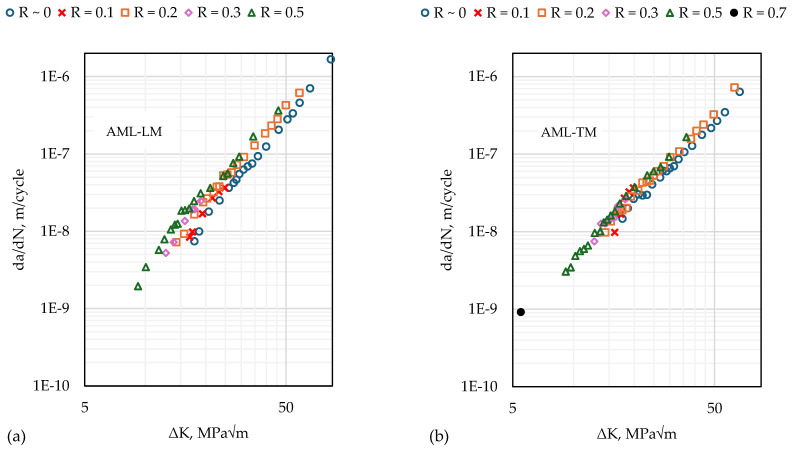
Fatigue crack growth rates for different load ratios; for (**a**) crack propagating longitudinally to the deposition direction and (**b**) crack propagating transversely to the deposition direction.

The outcomes of the fatigue testing are shown in Figure 7 for both directions. The testing range of ∆K corresponds to the so-called stage II (or Paris-regime) of fatigue crack growth stages. As expected, a higher R-ratio leads to higher fatigue crack growth rates at the same value of ∆K.

### 3.4. Crack Tip Opening Load Ratios 

The effect of the R-ratio as well as many other fatigue phenomena and effects can be explained with the plasticity-induced crack closure concept [40]. Crack closure implies that a fatigue crack remains closed for some portion of a tensile load cycle due to the formation of a plastic wake behind the crack tip. In other words, a certain force,$P_{op}$, has to be applied in order to open the crack and produce fatigue damage at its tip. In fatigue crack growth modelling, the simplest way to account for the crack closure phenomena is to redefine the crack driving force (which is the stress intensity factor range, ∆K), as the effective stress intensity factor range,

(5) $$∆K_{eff}=K_{max}-K_{op},$$

where $K_{op}$ is the opening stress intensity factor corresponding to the opening load $P_{op}$ shown in Figure 4 and Figure 5.

To correlate the FCG rates against this redefined crack driving force, $K_{op}$ needs to be determined for all R-ratios. $K_{op}$ can also be affected by many other parameters such as crack length, presence of stress concentration, and plate/specimen thickness. Therefore, progressive measurements of the opening stress intensity factor, $K_{op}$ (which is a function of the crack tip opening load, $P_{op}$) are required. These measurements, as mentioned in the previous Section, have been conducted using the compliance-based method and utilising the back face strain gauge. The methodology of the measurements has been discussed previously in this paper and general guidelines are provided in the ASTM standard [30]. 

It is also customary to present experimental results for the crack opening stress intensity factor or crack tip opening load in a dimensionless form by introducing the opening load ratio, U, defined as

(6) $$U=\frac{P_{max}-P_{op}}{P_{max}-P_{min}}=\frac{K_{max}-K_{op}}{K_{max}-K_{min}}$$

**Figure 8:**
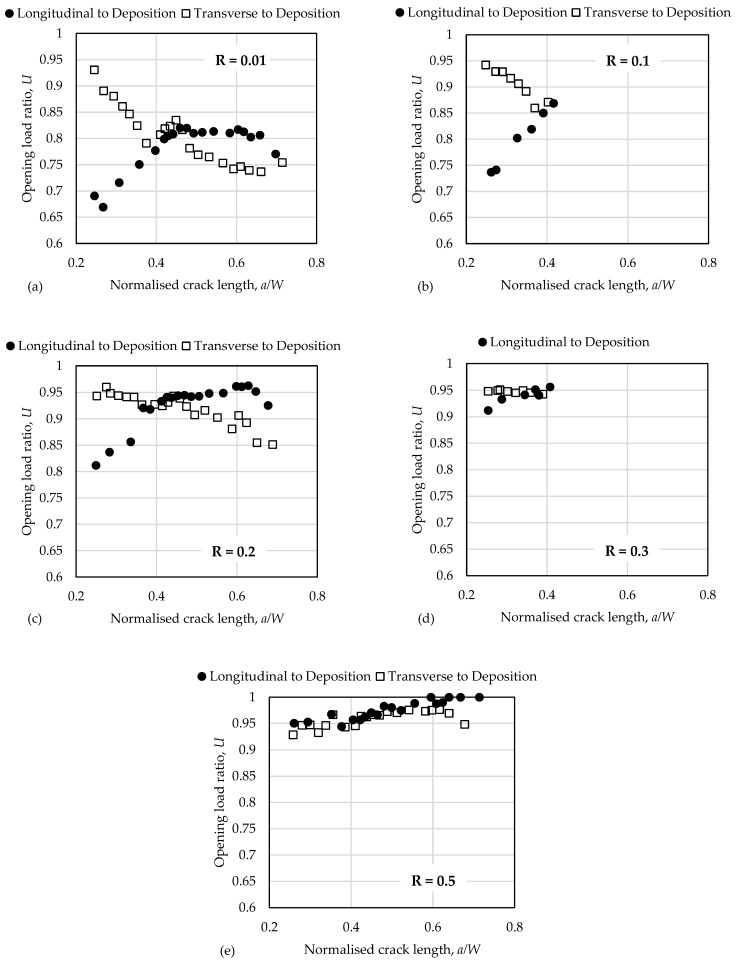
Fatigue crack opening ratio, U, as a function of normalised crack length, a/W when fatigue crack propagates longitudinally and transversely to the deposition direction for (**a**) R = 0.01, (**b**) R = 0.1, (**c**) R = 0.2, (**d**) R = 0.3, and (**e**) R = 0.5.

The crack closure effect becomes more pronounced at small values of the R—ratio and is almost negligible above R = 0.5. Therefore, no fatigue crack growth tests were conducted for R > 0.5. It can be seen from Figure 8 that the crack tip opening values are affected by the crack length, a, as the crack length changes the out-of-plane constraint conditions near the crack tip. Similar tendencies have been reported in previous studies for different materials and fatigue specimen dimensions.

The most notable observation is that the difference in the crack closure levels as quantified using the opening load ratio U between specimens oriented longitudinally or transversely is most significant for relatively small, normalised crack lengths (a/W < 0.4). Because the dominant portion of fatigue life is normally associated with the propagation fatigue cracks near its initial length, $a_{0}$, the initial differences in the crack tip opening values largely explain the significant difference in fatigue life of the specimens as demonstrated in Figure 3 and Figure 6. At longer crack lengths, crack closure is less pronounced and the difference in the closure level between the two directions is less distinguished. 

### 3.5. Crack Growth Rates versus Effective Stress Intensity Factor Range

**Figure 9:**
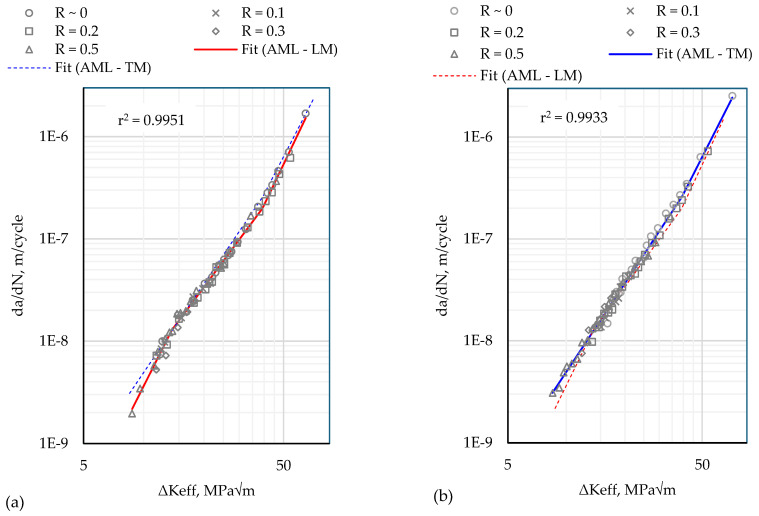
Crack growth rates, da/dN, versus the effective stress intensity factor range, $∆K_{eff}$, for crack propagating (**a**) longitudinally to the deposition direction, and (**b**) transversely to the deposition direction.

FCG (fatigue crack growth) rates in the longitudinal and transverse to the deposition directions are shown in Figure 9 after closure correction. The application of block loading resulted in multiple FCG rate curves with overlapping ranges of da/dN in Figure 7. After closure correction (Figure 9), the curves for different R-ratios collapse onto a single da/dN vs. ΔKeff curve. This outcome demonstrates the consistency of the experimental procedure since the multiple data points at any given da/dN in Figure 9 correspond to load blocks with different applied load ranges (ΔP) applied at different crack lengths. The block loading method enables multiple tests to be performed on the same specimen, avoiding the need to repeat the test on multiple specimens to validate the experimental method.

In accordance with previous FCG studies, e.g., [33], a piece-wise linear function comprising three linear segments is fitted to the da/dN vs. ΔKeff curve. This is to accommodate the changes in the slope at low (near threshold) and high (near fracture) crack growth rates. The coefficient of determination (r-squared value) of both fits is close to 1.

## 4. Discussion

This study reports fatigue properties for SDSS manufactured using the WAAM method and is an extension of previous work by the authors [19,20] by focusing on collecting and analysing FCG data. FCG rates and crack tip load opening ratios are provided for a broad range of loading parameters in terms of the stress intensity factor range ∆K as well as against $∆K_{eff}$, the stress intensity factor range correlated by crack opening values, for two directions: longitudinal to the deposition direction and in transverse to deposition direction. When expressed in terms of the “effective” stress intensity factor range, $∆K_{eff}$, the fatigue properties in both directions are almost identical, which demonstrates the significance of crack closure mechanisms for AM materials. Indeed, the fatigue crack tip opening ratio, U, reaches 0.65 for relatively small cracks and low R-ratios. 

This paper successfully applied a compliance-based technique in conjunction with strain gauge measurements for the evaluation of crack tip opening loads, $P_{op}$. The consistency of the evaluation improves significantly through increasing the fatigue crack length, a, which is also in a broad agreement with the previous studies. It is also demonstrated experimentally that $P_{op}$ changes significantly with the crack length and R-ratios. It is interesting to note that the tendencies of $P_{op}$ with an increase in the crack length are opposite for two crack propagation directions and a/W < 0.4. At larger ratios of a/W the crack tip opening loads are very similar for both directions. The effect of crack closure mechanisms on FCG rates vanishes for relatively high R-ratios, which is again in a general agreement with previous studies. The evaluated crack tip opening loads are sensitive to the choice of parameters used in the ASTM compliance-offset method. In the present work, we define the 2% offset relative to the mean compliance offset in the upper portion of the loading cycle leading to the achievement of better consistency in the experimental results. Therefore, one needs to be careful when comparing the present results with outcomes of similar studies for the same material.

In general, crack tip opening values (for relatively long cracks, which are not affected significantly by roughness-induced closure) are defined by the plastic properties of the material as well as the loading history. The yield properties and loading history for tested specimens are almost identical as reported in Section 3.1. Therefore, the large difference in the crack tip opening loads for two considered directions needs to be explained. 

It is believed that this difference in the crack tip opening values is due to the residual stresses induced using the rapid cooling nature of the WAAM bead layering process itself. Additively manufactured components can exhibit anisotropic material properties without the careful process control of cooling rates, parameters and post-process heat treatments, and therefore the residual stresses can be very different for different directions. Therefore, for relatively long cracks, in the presence of residual stresses, $K_{op}$ can be represented by two components: one is due to the plasticity-induced crack closure mechanism, $K_{picc}$, and the second component, $K_{res}$, is related to the residual stresses, or

(7) $$K_{op}=K_{picc}+K_{res}$$

The presence of residual stress and $K_{res}$ can explain the differences in $K_{op}$ and U between the longitudinal and transverse directions as well as a very different fatigue life of the specimens. Indeed, crack closure in the longitudinal direction is much more pronounced for relatively small cracks, which take a large portion of fatigue life. As a result, the specimen with fatigue crack propagation in the longitudinal direction has a much longer fatigue life than the specimen with a fatigue crack grown in the transverse to the deposition direction. However, to verify this hypothesis, one needs to simultaneously measure FCG rates and residual stresses, preferably on the same specimen, which may represent serious challenges. Indeed, values of $K_{res}$ for different specimens may be different as the residual stress can change significantly within the structure, in the present case with the WAAM manufactured test wall, as shown in Figure 1. Moreover, post fabrication processes such as machining are always associated with the release of residual stress, which again could present different values for across specimens. Therefore, the experimental verification of this hypothesis is left for future studies.

## 5. Conclusions

The main outcome of this research investigation is the experimentally measured FCG rates in terms of the effective stress intensity factor, $K_{eff}$. It was demonstrated that these rates are almost the same in two crack propagation directions: longitudinal and transverse to the WAAM deposition directions. This outcome essentially indicates the isotropy of the fatigue properties with respect to $K_{eff}$, although further research is required to verify this isotropy phenomenon in SDSS components produced using the WAAM process with different welding parameters. This finding could significantly reduce efforts, cost and time for the characterisation of the fatigue properties of AM materials.

Moreover, this research also demonstrates that crack growth rates are significantly affected by the plasticity-induced crack closure mechanism as well as residual stresses or residual stress intensity factors. The latter is possibly the reason behind the differences in the fatigue life of the specimen WAAM structures, and although this is consistent with reported findings, further investigations could provide confirmation. 

Overall, the current results and outcomes will help to facilitate the application of SDSSs fabricated using the wire-arc additive manufacturing process as load-bearing structural components and allow modern design, evaluation and maintenance methodologies (e.g., methods based on the damage tolerance concept [37]).

## Figures and Tables

**Table 1:** WAAM deposition parameters.

Parameters	
Droplet Transfer Mode	CMT
Contact tip to work distance (CTWD)	15.0 mm
Wire diameter	1.2 mm
Shielding gas	80% Ar + 20% CO_2_
Flow rate	20 L/min
Inter-pass temperature	100 °C
Wire-feed speed (WFS)	9.0 m/min
Travel speed (TS)	0.6 m/min
WFS/TS	15
Layer height (LH)	2.5 mm
Arc Energy	0.91 kJ/mm

## Data Availability

Data are contained within the article.
